# Parenting style and Chinese preschool children’s pre-academic skills: A moderated mediation model of approaches to learning and family socioeconomic status

**DOI:** 10.3389/fpsyg.2023.1089386

**Published:** 2023-02-06

**Authors:** Xiaoying Xia

**Affiliations:** ^1^School of Early Childhood Education, Shanghai Normal University Tianhua College, Shanghai, China; ^2^University of the Pacific, Stockton, CA, United States

**Keywords:** parenting style, approaches to learning, pre-academic skills, family SES, moderated mediation

## Abstract

This study examined the mediating role of children’s approaches to learning (ATL) in parenting style and Chinese preschoolers’ pre-academic skills (i.e., literacy and numeracy) as well as the moderating role of family socioeconomic status (SES) in the mediating process. Participants were 307 children aged five to six years old from four public kindergartens in Shanghai, China. Parents provided demographic information and reported their parenting style (i.e., authoritative, authoritarian, and permissive) and teachers rated each child’s ATL and pre-academic skills. Results indicated that: (1) authoritative parenting positively related to children’s pre-academic skills while no significant relationships were identified either for authoritarian or permissive parenting with pre-academic skills; (2) children’s ATL partially mediated the relationship between authoritative parenting and children’s pre-academic skills; (3) family SES moderated the relationship between children’s ATL and pre-academic skills. Specifically, children’s ATL was more strongly related to pre-academic skills for children from low SES families as compared to their high-SES peers. These findings contribute to the understanding of the effects of parenting styles on Chinese children’s early academic achievement and underscore the importance of ATL to children’s pre-academic skills, especially for low-SES children.

## 1. Introduction

Transitioning from preschools to primary grades represents a critical life milestone for young children as they are required to be academically and social–emotionally ready to function well in formal schooling (Hair et al., 2006). Children’s pre-academic skills, including emergent literacy and numeracy skills, are essential ingredients of school readiness (Jeon et al., 2020). Research suggests that children’s early academic skills at school entry are consistent and strong predictors of later academic achievement (Duncan et al., 2007; Davies et al., 2016). However, the development gap in children’s academic achievement has appeared as early as kindergarten, and studies have shown that the academic skills of children from low-income families are significantly lower than that of their peers from high-income families (Zhang et al., 2019). In order to narrow the gap in children’s academic readiness for school, it is necessary to explore the factors that affect the development of children’s early academic skills.

Parenting style has reoccurred in the literature as influential for young children’s development, nevertheless most studies have sampled Western populations and relatively little is known about the associations between parenting style and preschool children’s pre-academic skills in the non-Western contexts, like China. In addition, despite the evidence for the link between parenting styles and children’s academic achievement, relatively little empirical work has been conducted to uncover the mechanism underlying this relationship. Some scholars have speculated that the pathway from parenting to academic achievement can be accounted for by “academic enablers” (DiPerna, 2006), including children’s motivation, engagement, and learning behaviors. These attitudes and behaviors in learning are conceptualized as approaches to learning (ATL; Li-Grining et al., 2010). ATL has been documented as a significant contributor to children’s academic achievement in many studies (e.g., Schmerse, 2020), however, empirical work examining the mediating role of ATL in the pathway from family factors to children’s academic achievement is relatively limited. Moreover, prior research suggests that children’s ATL varies across diverse socioeconomic status (SES) families (Buek, 2019). However, the question remains unclear whether ATL functions similarly or differently across children of different SES families. If low-SES children reap more benefits from ATL, promoting children’s ATL would be an effective way to mitigate the SES-based achievement gap. If high-SES children reap more benefits from ATL, ATL would be viewed as an important factor for the widening achievement gap. Therefore, this study aimed to investigate whether parenting style was associated with Chinese children’s early academic skills and to further explore whether this relation was mediated by children’s ATL and whether family SES moderated the mediating process.

### 1.1. Parenting style and children’s pre-academic skills

Parenting style is conceptualized as a constellation of attitudes and behaviors that parents display in rearing their children (Darling and Steinberg, 1993). In Baumrind’s (1967) seminal work, three types of parenting style were proposed: authoritarian, authoritative, and permissive. Authoritarian parents, characterized by high demandingness and low responsiveness, have high expectations for children’s obedience and respect for parental authority and show limited responsiveness to children’s needs. Authoritative parents, characterized by high responsiveness and high demandingness, tend to set rules for children and rely on reasoning and negotiations with children to enforce rules. Permissive parents are high on responsiveness but low on demandingness. They set few limits on their children’s behaviors and show high responsiveness to children’s needs and wants.

Based on bioecological systems theory, parent–child interactions are of critical importance to children’s acquisition of early academic skills (Bronfenbrenner and Morris, 2006). A large body of empirical research suggests that authoritative parenting positively and authoritarian and permissive parenting negatively associated with children’s academic achievement [see a review by Pinquart (2016)]. However, most of the extant research has been conducted in the West and studies sampling non-Western populations report inconsistent conclusions. For example, authoritarian parenting was identified to be positively related with Chinese adolescents’ academic achievement (Leung et al., 1998) and permissive parenting was reported as more beneficial for Spanish adolescents’ school outcomes than authoritative parenting (Garcia and Gracia, 2009). As parenting styles could be interpreted differently across cultures, the effect of parenting style on children’s school outcomes might be different accordingly. In the Chinese context, the existing studies regarding parenting style have primarily focused on primary and secondary school students (Xie et al., 2022), and research sampling preschool children has mostly focused on the effect of parenting styles on children’s emotional and social development (Lin et al., 2022). Furthermore, children of different age groups have different developmental needs. There is some evidence that specific parenting dimensions have differential effects on children’s academic performance across developmental stages. For example, Zhu and Liu (2019) found that authoritative parenting has a stronger positive effect on 6–9 years old children’s reading score, while permissive parenting has a stronger positive effect on 10–15 years old adolescents’ reading performance.

Research on the effects of parenting style on Chinese preschool children’s academic achievement is relatively limited and the few extant studies tend to be less consistent. For example, Ren and Edwards (2017) found a positive correlation between authoritative parenting and preschool children’s early academic skills, while Li et al. (2016) failed to find a significant correlation between the two variables. Another study sampling Chinese American preschool children also reported an insignificant link between parenting style (i.e., authoritative and authoritarian) and academic achievement (Chen et al., 2015). They believed that parenting style functioned more by providing emotional family atmosphere, and have minimal effects on children’s academic performance (Chen et al., 2015). In view of the limited research in the Chinese context, the contribution of different parenting dimensions to preschool children’s pre-academic skills requires further empirical research.

### 1.2. The mediating role of children’s approaches to learning

Parenting style constitutes an environmental factor for children’s development and may play an indirect role *via* children’s personal characteristics (Xie and Li, 2022). DiPerna (2006) proposed that “academic enablers” such as motivation, academic engagement and learning behaviors are important contributors to children’s academic achievement. Children’s approaches to learning (ATL) refers to positive learning attitudes and behaviors that children display in learning activities, including persistence, sustained focus, initiative, planning, and cooperative engagement in group learning (McCWayne et al., 2004). As ATL is closely involved in the process of learning, it has been consistently shown as an important predictor of children’s learning outcomes. For example, McCWayne et al. (2004) sampled US Head Start children and found ATL significantly predicted preschool children’s language and math skills. A longitudinal study by Vitiello and Greenfield (2017) found that preschool children with higher levels of ATL performed better in math and reading than those with lower ATL when were in the first grade of primary school. Research sampling Chinese preschool children has found similar conclusions. For example, Zhang and Zhou (2018) found that children’s ATL such as self-control, persistence, curiosity and interest significantly predicted preschool children’s language development and math skills.

ATL is a developmental characteristic and parenting style is foundational for the formation of ATL in children as it provides an emotional environment for parent–child daily interactions. Western studies have found that supportive parenting positively predicts preschool children’s learning interest and persistence, while corporal punishment and authoritarian parenting can damage children’s learning motivation, which in turn affects their persistence in tasks and learning strategies (Martin et al., 2013). Research on Chinese preschool children also found that parenting styles affect the development of preschool children’s ATL. Specifically, authoritative parenting style positively predicted, while authoritarian parenting style negatively predicted children’s ATL (Ye et al., 2021). Based on the literature above, ATL might be an important mechanism by which parenting styles affect children’s early academic performance.

### 1.3. The moderating role of family socioeconomic status

Family income, parents’ educational levels and occupational prestige are important indicators of family socioeconomic status (SES) and have been consistently documented to relate to children’s academic achievement (Conger and Donnellan, 2007). Children from low-SES families tend to score lower on academic skills than their higher-SES peers (Ip et al., 2016). The academic achievement gaps between high and low-SES children have emerged as early as preschool years. Prior research has suggested that ATL can work as a protective factor for children in the context of adversity. However, Luthar et al. (2000) claimed that children’s early ATL skills can serve as resilience for children who experience risk factors and they argued that high-risk children benefited more from ATL than low-risk children. Also, there is some empirical evidence in the literature that ATL may function as a source of resilience for children facing risks. For example, Razza et al. (2015) investigated children from low-income families in the United States and claimed that children’s ATL is particularly beneficial for improving the academic skills of at-risk children. In the study, they primarily sampled low-income children and did not investigate the relationship between ATL and children’s academic achievement across diverse family SES. Shah et al. (2018) sampled 6,200 U.S. preschool children and explored the interaction between family SES and children’s curiosity in shaping children’s academic achievement. They found that children’s curiosity positively predicted their reading and math performance, and the association was stronger among children from lower SES families. As children’s curiosity constitutes an important element of ATL, it can be hypothesized that family SES would moderate the relationship between ATL and pre-academic skills. In view of this, the moderating role of family SES in the relationship between children’s ATL and their pre-academic skills will be explored among Chinese preschoolers in the present study.

### 1.4. The present study

**Figure 1:**
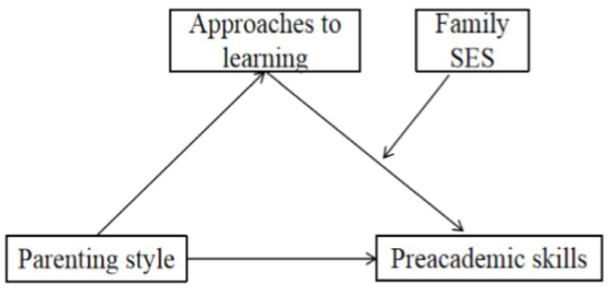
The moderated mediation model.

As reviewed above, previous work has primarily investigated the direct effect of parenting styles on the academic achievement among middle school and primary school-age children in the Western context. However, the effects of parenting styles on preschool children’s pre-academic skills received less attention. In addition, the mechanism underlying the relationship between parenting styles and children’s academic performance was not adequately addressed. ATL is documented to closely relate to children’s pre-academic skills, but less work is conducted to explore whether this relationship varies across diverse SES families. In view of this, this study considers both family variables and child variables to test a moderated mediation model by examining the mediating role of ATL in parenting style (i.e., authoritative, authoritarian, and permissive) and children’s pre-academic skills as well as the moderating role of family SES in the mediating process. The hypothesized model is presented in Figure 1. To test the model, the following hypotheses were proposed: (1) authoritative parenting positively related to children’s pre-academic skills (Hypothesis 1); (2) authoritarian and permissive parenting negatively related to children’s pre-academic skills (Hypothesis 2); (3) children’s approaches to learning mediated the relationship between parenting style and children’s pre-academic skills (Hypothesis 3); (4) family SES moderated the relationship between children’s approaches to learning and pre-academic skills (Hypothesis 4).

## 2. Materials and methods

### 2.1. Participants

In this study, a clustered random sampling method was used to elicit participants from four public kindergartens in Shanghai, China, including one model kindergarten, one first-class kindergarten and two second-class kindergartens. In general, model kindergartens and first-class kindergartens have more children from higher SES families while second-class kindergartens include more children from lower SES children. Chinese public kindergartens provide services for three to six years old children and the present study focused on five to six years old children in senior classes of kindergartens. A total of 341 questionnaires were distributed among the target population and 324 respondents agreed to participate in the study and returned the family demographic and parenting questionnaires, resulting in a return rate of 95%. List-wise deletion method was used to delete cases with missing values. Finally, a sample of 307 parents and their children from 11 classrooms of the four kindergartens were involved in this study. Twenty two teachers of the child participants were invited to rate children’s school readiness based on daily observations.

The child participants in this study ranged from five to six years old (Mean = 5.53, SD = 0.50) with five years olds accounting for 46.9% (*n* = 144) and 6 years olds accounting for 53.1% (*n* = 163). The proportions of boys and girls were balanced, among which 154 were girls, accounting for 50.2% (*n* = 154). The majority children in this study were singletons, accounting for 83.4% (*n* = 256). As regards parent participants, the mean age is 35.5 years (SD = 3.6) with two thirds respondents being mothers (65.1%, *n* = 200). Approximately 84% (*n* = 258) of the population obtained a college degree or above, suggesting that the majority participants were relatively well-educated. The vast majority of families are intact families, accounting for 97.7% (*n* = 300). According to the statistics reported by Shanghai Municipal Statistics Bureau (2015), the average *per capita* disposable income for each household was *RMB* 3,975 (*$*576) a month. In terms of family economic status, 31.3% (*n* = 96) families earned a monthly income below *$*1,450; 53.3% (*n* = 163) families reported a monthly income ranging from *$*1,450 to *$*4,350; 15.7% (*n* = 48) families had a monthly income higher than *$*4,350.

### 2.2. Measures

#### 2.2.1. Family socioeconomic status

In the present study, family monthly income, parents’ educational levels, and occupational prestige were included as indicators for family SES. The parent participants reported diverse levels of family monthly income, ranging from *$*427 and below (coded as 1), *$*427–853 (coded as 2), *$*853–1,422 (coded as 3), *$*1422–4,266 (coded as 4), to *$*4266–7,110 (coded as 5), and to *$*7,110 and above (coded as 6). In addition, parents’ educational levels were assessed on a 5-point scale, from junior middle school and below (coded as 1), secondary school (coded as 2), three year college (coded as 3), four year college (coded as 4) to graduate programs and above (coded as 5). Also, parents’ occupation was evaluated on a 3-point scale and ranged from farmers, migrant workers, service workers (coded as 1), foreign enterprise workers, general enterprise workers, and freelancers (coded as 2) to civil servants, professional and technical personnel, enterprise management personnel, cultural, educational and scientific personnel (coded as 3). A composite score of the family SES was computed using principal component analysis based on the following formula: Family SES = (*β*1*Z parental education+*β*2*Z parental occupation+*β*3* Z family income)/*εf*, with *β*1-3 as the factor loadings and *εf* as the eigenvalue for the first factor (OECD, 2012). In general, higher SES scores represent higher family SES in the present study.

#### 2.2.2. Parenting style

Parenting style was measured with the Parenting Styles and Dimensions Questionnaire (PSDQ; Robinson et al., 2001). The PSDQ is comprised of 32 items and uses a 5-point Likert format (1 = never, 2 = once in a while, 3 = about half of the time, 4 = very often, 5 = always) to assess how frequent parents display certain attitudes and behaviors in rearing their children. There are three dimensions in the PSDQ: authoritative parenting (15 items), authoritarian parenting (12 items), and permissive parenting (5 items). The authoritative parenting evaluates how frequent parents demonstrate warmth and acceptance, reasoning and induction, and democratic participation in parent–child interactions. The sample items include, “take child’s desire into account before asking him/her to do something.” The authoritarian parenting evaluates the frequency of parents’ use of physical coercion, verbal hostility, and non-reasoning and punitive strategies. The sample items include, “guide child by punishment more than by reason.” The permissive parenting measures how frequent parents show permissiveness in rearing children. The sample items include, “I state punishments to my child and do not actually do them.” The mean of each parenting dimension was calculated by averaging all the items in that specific dimension. Higher scores indicated a higher frequency of a specific type of parenting. In this sample, the Cronbach’s alphas for authoritative, authoritarian, and permissive parenting were 0.87, 0.76, and 0.62, respectively.

#### 2.2.3. Children’s approaches to learning

Children’s learning behavior was measured with the approaches to learning sub-domain of the social competence subscale from the Early Development Instrument (EDI; Janus and Offord, 2007). The EDI consists of 103 items and assesses children’s early development in physical health, language and cognitive development, social competence, emotional maturity, and communication skills and general knowledge. The Chinese version of the EDI has been validated in Chinese preschool children and presented desirable psychometrical soundness (Ip et al., 2013). The ATL subdomain of the EDI contains 9 items and measures children’s learning behaviors such as working independently, following directions and class routines, and adjusting to changes. Sample items include “follows directions” and “works independently.” Questions in the ATL measure were answered on a 3-point scale, ranging from often or very true, sometimes or somewhat true, to never or not true. For data analysis purposes, all responses on 3-point Likert-scale items were coded 0, 5, and 10 (Janus and Offord, 2007). The average score in this domain was calculated to represent children’s ATL. Higher scores indicate stronger ATL for children. The Cronbach alpha coefficient was 0.95 for the measure of ATL in the current sample.

#### 2.2.4. Children’s pre-academic skills

Children’s pre-academic skills were measured using items from the language and cognitive development subscale of the EDI (Janus and Offord, 2007). The language and cognitive development subscale consists of 26 items and assesses children’s basic and advanced literacy skills, basic numeracy skills and interest in literacy/numeracy and memory. It is noted that the subdomain of interest in literacy/numeracy and memory was not included for further analysis as it was not applicable for the measure of pre-academic skills. Finally, 21 items in the language and cognitive development subscale were used to measure children’s pre-academic skills. Basic literacy assesses children’s basic abilities to recognize written words and to participate in literacy-oriented activities. Sample items include “knows how to handle a book” and “is able to write his or her name in Chinese.” Advanced literacy assesses children’s emergent reading and writing. Sample items include “is able to read simple words” and “is interested in writing voluntarily.” Basic numeracy assesses children’s number recognition and counting, and basic mathematical concepts. Sample items include “sort and classify objects by a common characteristic” and “recognize geometrical shapes.” All answers to the questions in these measures were scored on a 2-point scale: “yes” if a child possesses a skill and “no” if he or she does not (Janus and Offord, 2007). For data analysis purposes, all responses on binary items were coded 0 or 10. The mean of all items in the basic literacy, advanced literacy and basic numeracy subdomains was calculated to represent children’s pre-academic skills. The Cronbach alpha coefficient was 0.83 for the measure of pre-academic skills.

### 2.3. Data collection

After obtaining approval from the IRB in the university, the investigator contacted the principals of the potential kindergartens and finally obtained permission from four kindergartens in Shanghai, China. Parent participants were elicited by distributing letters when they came to school to pick up their children. If they were willing to participate, they signed an informed consent form and give permission for the investigator to access their children’s data. One parent of the child was asked to fill out a survey questionnaire containing items about their family background information and parenting style. The parent surveys were collected by the investigator the next day. Subsequent to collecting parent data, teachers, who were familiar with the child participants, were invited to rate children’s approaches to learning and pre-academic skills based on their daily observations. To reduce measurement errors, all the teacher participants received two rounds of measurement training based on the training materials provided by Offord Centre for Child Studies (i.e., the developer of the EDI). Each participating teacher was given a teachers’ guide on the use of the EDI assessment forms in which the assessment criteria for all the items were clarified and children’s exemplary performance and behaviors were also presented in the training sessions. Teachers rated children’s ATL and pre-academic skills based on their daily observations and personal interactions with children.

### 2.4. Data analysis

At first, means, standard deviations and bivariate correlations of the main variables were calculated using SPSS 26.0. Second, a sequential multiple regression was conducted to determine the relationship between parenting style on children’s pre-academic skills while controlling for children’s age, gender, with siblings or not, and family SES. The *β* value (standardized regression coefficient) was referred to determine the effect of each type of parenting style on pre-academic skills. A statistically significant *β* value would indicate the potential influence of specific type of parenting. Third, Model 4 and Model 14 in PROCESS Macro (Hayes, 2017; Version 3.3) were employed to test the mediating role of ATL and the moderated mediating role of family SES in parenting styles and children’s pre-academic skills, respectively. The effects are assumed as statistically significant when the lower limit (Boot LLCI) and upper limit (Boot ULCI) of bootstrap results in 95% confidence interval are either below or above zero (Hayes, 2017).

## 3. Results

### 3.1. Descriptive analysis

Zero-order correlations, means, and standard deviations of the main variables were presented in Table 1. Correlational analyzes showed that authoritative parenting positively related to children’s ATL (*r* = 0.20, *p* < 0.01), and pre-academic skills (*r* = 0.23, *p* < 0.001). Authoritarian parenting was negatively related to children’s pre-academic skills (*r* = −0.12, *p* < 0.05) and permissive parenting negatively associated with children’s ATL (*r* = −0.12, *p* < 0.05). Children’s ATL was highly linked with their pre-academic skills (*r* = 0.51, *p* < 0.001). In addition, family SES positively related to authoritative parenting (*r* = 0.27, *p* < 0.001), but negatively related to authoritarian (*r* = −0.15, *p* < 0.01) and permissive parenting (*r* = −0.13, *p* < 0.05). These results suggest that parents from higher SES families reported higher levels of authoritative parenting but lower levels of authoritarian and permissive parenting. In addition, family SES positively related to children’s early academic skills (*r* = 0.24, *p* < 0.01) and no significant relationship was identified for children’s ATL with family SES.

**Table 1:** Means, standard deviations, and zero-order correlations of main variables.

Variables	2	3	4	5	6
1. Family SES	0.27***	−0.15**	−0.13*	0.04	0.24***
2. Authoritative parenting	–	−0.33***	−0.32***	0.20**	0.23***
3. Authoritarian parenting		–	0.44***	−0.10	−0.12*
4. Permissive parenting			–	−0.12*	−0.07
5. Approaches to learning				–	0.51***
6. Pre-academic skills					–
*M*	3.98	2.00	2.36	8.32	8.20
SD	0.52	0.42	0.65	2.17	1.64

*n* = 307, *p < 0.05, **p < 0.01, ***p < 0.001.

A series of *t*-tests were conducted to determine whether parenting style, children’s approaches to learning and pre-academic skills vary by children’s gender, age, and only-child status as these variables were evidenced to be related to either parenting style or children’s development outcomes. Statistics showed that there were significant gender differences in children’s ATL. Girls scored significantly higher than boys (*t* = −3.40, *p* < 0.01) in ATL. Also, age difference in children’s pre-academic skills was identified and 6-year-old children were significantly higher than that of 5-year-old children (*t* = −2.12, *p* < 0.05). There are significant differences in the authoritative and permissive parenting between only child and children with siblings. Parents of only child reported higher authoritative parenting then those with more than one children (*t* = 3.15, *p* < 0.01), whereas parents with more than one children reported higher levels of permissive parenting than those with one children (*t* = −2.02, *p* < 0.05). In view of the effects of children’s gender, age, only child status on the main research variables, they were used as control variables in the follow-up analysis.

### 3.2. Parenting style and children’s pre-academic skills

To test the relationship between three types of parenting styles and children’s pre-academic skills, a sequential multiple regression with children’s pre-academic skills as the dependent variable, and the three types of parenting styles as independent variables was conducted after controlling for child and family demographic variables. Multiple regression analysis (see Table 2) showed that demographic factors such as children’s gender, age, and whether they were only child or not and family SES altogether, explained 8.6% of the variation in children’s pre-academic skills (*ΔR^2^* = 0.086, *F* [4,302] =7.07, *p* < 0.001). Family SES positively related to children’s pre-academic skills (*β* = 0.25, *p* < 0.01), and children from high-SES families scored higher in pre-academic skills than children from low-SES families. Three types of parenting styles explained a 3.6% variation in children’s pre-academic skills (*ΔR^2^* = 0.036, *F* [3,299] = 4.13, *p* < 0.01). Moreover, among the three types of parenting dimensions, authoritative parenting significantly and positively associated with children’s early academic skills (*β* = 0.19, *p* < 0.01), while authoritarian parenting and permissive parenting did not significantly relate to children’s pre-academic skills with control variables included. Thus, hypothesis 1 was supported, and hypothesis 2 was rejected in the present study.

**Table 2:** Sequential multiple regression of pre-academic skills on parenting style.

	*B*	SE *B*	*β*	*ΔR^2^*	*F*	*t*	*p*
Step 1				0.086***	7.07		0.000
Child gender	0.35	0.18	0.11			1.94	0.053
Child age	0.45	0.18	0.14*			2.47	0.014
Only child status	0.18	0.26	0.04			0.68	0.498
Family SES	0.14	0.03	0.25***			4.24	0.000
Step 2				0.036**	4.13		0.003
Authoritative	0.61	0.19	0.19**			3.66	0.000
Authoritarian	−0.14	0.25	−0.04			0.57	0.572
Permissive	0.06	0.16	0.02			0.37	0.710

*n* = 307, ***p < 0.001; **p < 0.01, *p < 0.05.

### 3.3. Approaches to learning as a mediator

Model 4 in PROCESS Macro (Hayes, 2017; Version 3.3) was used to test the mediating role of children’s ATL in the relationship between authoritative parenting and pre-academic skills. Authoritative parenting positively predicted children’s pre-academic skills (*β* = 0.24, *t* = 4.29, *p* < 0.001) and children’s ATL (*β* = 0.23, *t* = 4.04, *p* < 0.001). Also, children’s ATL positively related to their early academic skills (*β* = 0.47, *t* = 9.20, *p* < 0.001). After adding the mediating variable of ATL to the model, the predictive effect of authoritative parenting on children’s academic skills is still significant (*β* = 0.13, *t* = 2.64, *p* < 0.01), which indicates that authoritative parenting can not only directly predict children’s early academic skills, but also indirectly predict children’s early academic skills through ATL. Children’s ATL plays a partial mediating role in authoritative parenting and children’s early academic skills, supporting research hypothesis 3. The direct effect, indirect effect and total effect of authoritative parenting on children’s early academic skills were shown in Table 3. The total effect was 0.24 and the indirect effect was 0.11. Altogether, the indirect effect accounted for 45.83% of the total effect. This result suggests that ATL mediated 45.83% of the effect of authoritative parenting on children’s pre-academic skills.

**Table 3:** Total effect, direct effect, and indirect effect.

	Effects	Boot (SE)	Boot LLCI	Boot ULCI
Total effect	0.24	0.06	0.13	0.35
Direct effect	0.13	0.05	0.03	0.23
Indirect effect	0.11	0.04	0.05	0.19

*n* = 307.

### 3.4. The moderated mediating model

In order to examine the moderating effect of family SES in the mediation model, Model 14 in PROCESS Macro (Hayes, 2017; Version 3.3) was used to test the hypothesized moderated mediation model with children’s gender, age, and only child status as control variables, pre-academic skills as the outcome variable, authoritative parenting as the predictor, ATL as the mediating variable, and family SES as the moderating variable. The interaction between family SES and children’s ATL significantly contributed to children’s early academic skills (*β* = −0.05, *t* = −3.26, *p* < 0.01). The moderated mediation index was −0.01, and the 95% Bootstrap confidence interval was [−0.019, −0.003], excluding 0. These results suggest that the moderated mediation model was validated, supporting hypothesis 4 proposed in this study. Family SES moderated the effect of ATL on children’s pre-academic skills, and then moderated the mediating effect of ATL on authoritative parenting and children’s pre-academic skills.

**Figure 2:**
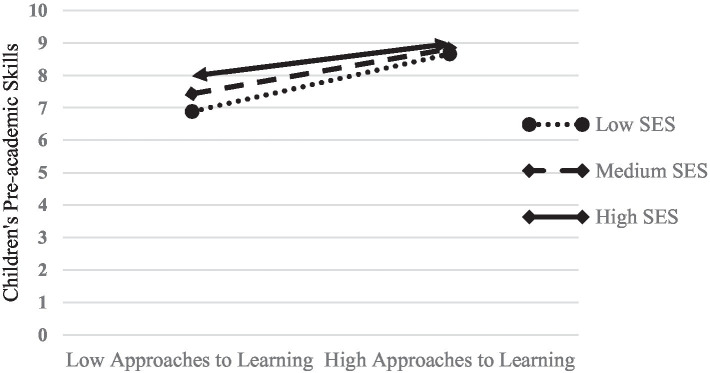
The moderating role of family SES on approaches to learning and pre-academic skills.

To further probe the interactions between family SES and children’s ATL, conditional regression lines of children’s pre-academic skills on ATL were plotted for children at one standard deviation below, the mean, and one standard deviation above for family SES (Aiken and West, 1991), labeled as low, medium, and high SES in Figure 2. Regardless of children’ family SES levels, children’s ATL all positively related to their pre-academic skills with a stronger relationship for children from low SES families. Among the three levels of family SES, ATL exerted the largest mediating effect for low SES families [*β* = 0.13, Boot CI 95% (0.05, 0.22)]. The mediating effect of children’s approaches to learning is relatively weaker for medium SES families [*β* = 0.10, Boot CI 95% (0.04, 0.17)] and the weakest for high-SES families [*β* = 0.07, Boot CI 95% (0.03, 0.13)].

## 4. Discussion

### 4.1. Parenting style and children’s pre-academic skills

This study found that authoritative parenting positively related to children’s pre-academic skills after controlling for child and family demographics. This finding is in line with previous literature that authoritative parenting positively contributed to preschool children’s academic skills and language development (Bingham et al., 2017). Also, a meta-analysis, primarily focused on parenting style of Chinese adolescents, reported that positive parenting behaviors such as emotional warmth, autonomy and support positively linked with adolescents’ academic achievement (Xie et al., 2022). According to self-determination theory (SDT; Deci and Ryan, 1985), children’s intrinsic motivation for learning stems from the satisfaction of their psychological needs for autonomy, competence and belonging. In the daily interactions, authoritative parents create affective and secure family environment by showing support, respect, understanding and caring for children, which in turn may motivate children to participate more actively in learning activities and acquire better pre-academic skills.

Although correlation analysis indicated that authoritarian parenting negatively correlated with children’s pre-academic skills, its effect on pre-academic skills was not identified after controlling for demographic variables and other types of parenting style (i.e., authoritative and permissive parenting). This result is in contrast to many studies conducted in the West. For example, Tamis-LeMonda et al. (2004) reported that authoritarian behaviors such as interference and harsh punishment could damage parent–child relationships, thereby negatively affecting children’s development of pre-academic skills. Previous research sampling Chinese populations has mostly focused on adolescents and reported a negative link between authoritarian parenting and academic achievement (Chen et al., 2015; Xie et al., 2022). Nonetheless, a recent study focusing on Chinese preschoolers revealed that the sub-dimensions of authoritarian parenting (i.e., verbal hostility and non-reasoning/punitive strategies) had differential effects on children’s school outcomes (Li and Xie, 2017). Specifically, they found negative effects of parents’ non-reasoning/punitive strategies but positive effects of parents’ verbal hostility on children’s language, literacy, numeracy and science skills. As a global parenting construct of authoritarian parenting was adopted in the present study, the conflicting effects among the various sub-dimensions of authoritarian parenting might hinder the possibility to identify significant effects. In addition, permissive parenting was not found to relate to children’s pre-academic skills. It should be noted that the low internal consistency reliability of permissive parenting in this study may limit its ability to exert significant effects on children’s pre-academic skills.

### 4.2. The mediating role of approaches to learning

As expected, this study found that authoritative parenting positively related to ATL, which in turn positively associated with children’s pre-academic skills. Authoritative parents, characterized by autonomy and support, tend to allow children to explore their environment, encourage young children to solve problems independently and involve children in the process of decision making. All of these behaviors are conducive to the development of ATL (Jeon and Neppl, 2019). In contrast, restrictive and harsh parenting behaviors will inhibit children’s exploratory behavior and hinder the development of positive learning behaviors (Larzelere et al., 2013). Moreover, in this study ATL was found to positively relate to pre-academic skills. This result is consistent with prior literature that preschool children’s ATL significantly predicted their early language and math skills (Zhang and Zhou, 2018). McDermott et al.’s (2014) longitudinal study of preschool children also showed that children with lower levels of motivation and focused attention in preschools performed worse in different academic subjects when they were in the first grade of primary schools. It is possible that children with higher levels of ATL can maintain focus, follow instructions, and inhibit certain behaviors in learning settings so that they can make better use of learning opportunities and develop stronger academic skills (Schmerse, 2020). In contrast, children with poor ATL are easily distracted and lack perseverance in tasks, which will hinder the development of children’s early academic skills (Beisly et al., 2020).

Results from the present study showed that children’s ATL partially mediated the relationship between authoritative parenting and children’s pre-academic skills. Authoritative parenting can not only directly related to children’s early academic skills, but also indirectly related to pre-academic skills *via* ATL. This result supports Hill and Palacios’ (2020) study with Early Childhood Longitudinal Study-Kindergarten Cohort (ECLS-K) 2011 longitudinal data, which found that parents’ warmth and support helps promote children’s ATL, which in turn facilitates children’s acquisition of reading skills. Authoritative parenting characterized by warmth and support, reasoning guidance, and democratic participation helps to create a positive and democratic family atmosphere, which is conducive to children’s development of ATL. And enhanced ATL directly affects children’s learning process and promotes the acquisition of academic skills.

### 4.3. The moderating role of family socioeconomic status

The relationship between ATL and children’s early academic skills was moderated by family SES in this study. Although in different SES families, children with higher levels of ATL display better academic skills, the relationship was stronger for children from lower SES families. It can be seen that ATL is an important protective factor for low-SES children’s the development of academic skills. This result supports a prior study by Shah et al. (2018), who found that family SES moderated the relationship between curiosity and preschool children’s reading and math skills, and this relationship was stronger for low-SES children. Also, Razza et al. (2010) claimed that ATL acted as a protective factor for economically disadvantaged children who are at risk in school readiness measures. The greater effects of ATL on children’s pre-academic skills might be because children from low SES families have less access to home educational resources and the drive for academic achievement is closely related to children’s ATL (Deci and Ryan, 1985). In the context of scarce educational resources, the way how children interact with the environment and peers and the initiative and activeness they display in learning activities play a more important role in children’s acquisition of academic skills. High levels of approaches to learning can help children cope with the disadvantages associated with low SES family environment.

## 5. Implications

This study contributes to the current literature by examining the mechanism underlying the relationship between parenting style and Chinese children’s pre-academic skills. The results of the study have several important implications. At first, we found that authoritative parenting made a positive contribution to children’s early academic skills. Parents are encouraged to adopt authoritative parenting and tend to be responsive to children’s feelings or needs, respect children’s opinions, and give explanations for obeying certain rules and consider children’s wishes in family affairs. The respect, understanding, and care provided by parents helps create benevolent family atmosphere and facilitate children’s development of early academic skills. Second, authoritative parenting positively related to children’s pre-academic skills *via* ATL and this mediating effect was stronger for low-SES children. As ATL positively related to children’s pre-academic skills, parents should attach importance to ATL and try to cultivate desirable learning behaviors when they are involved in children’s education. At present, many parents put more emphasis on children’s academic achievement than on children’s ATL. The preschool years constitute a critical period for children’s development of ATL. Thus, parents should adopt positive and democratic parenting behaviors to enhance children’s attention, persistence, independence and other good learning behaviors, thereby promoting the acquisition of early academic skills. Kindergartens can also improve teachers’ knowledge and understanding of ATL and conduct workshops and specialized trainings for teachers on how to stimulate children’s curiosity in activities, develop children’s persistence in tasks, and guide children to actively explore and solve problems independently. Third, for economically underprivileged children, parents need to enhance communications with their children and try to adopt authoritative parenting, which is beneficial for the development of ATL and pre-academic skills as well.

## 6. Limitations and suggestions for further studies

Despite its contributions to the literature, several limitations need to be acknowledged in this study. At first, as parenting style was assessed only through parental self-reports, parental bias might be created as a result of social desirability. More accurate information can be obtained by direct observations of parent–child interactions in the family context. Second, this study used a cross-sectional design to measure ATL and children’s pre-academic skills. In the future, longitudinal designs could be used to further validate the mediating role of ATL in family variables and children’s academic outcomes. Third, in order to include diverse SES populations, the study elicited parents from four public kindergartens based on different levels of family SES. As the participants were all from one economically developed city of China (i.e., Shanghai), the conclusions cannot be generalized to other Chinese regions. Future research can increase sample size to include children from both economically developed and backward regions in China to replicate this study. Last but not the least, this study does not distinguish between mothers and fathers, and whether maternal and paternal parenting styles have different effects on children’s ATL and early academic skills also deserves further investigation.

## Data availability statement

The original contributions presented in the study are included in the article/supplementary material, further inquiries can be directed to the corresponding author.

## Ethics statement

The studies involving human participants were reviewed and approved by University of the Pacific. Written informed consent to participate in this study was provided by the participants’ legal guardian/next of kin.

## Author contributions

XX collected and analyzed the data, and wrote the manuscript independently.

## Conflict of interest

The author declares that the research was conducted in the absence of any commercial or financial relationships that could be construed as a potential conflict of interest.

## Publisher’s note

All claims expressed in this article are solely those of the authors and do not necessarily represent those of their affiliated organizations, or those of the publisher, the editors and the reviewers. Any product that may be evaluated in this article, or claim that may be made by its manufacturer, is not guaranteed or endorsed by the publisher.
